# Intensive Care Unit Nurses’ Professional Autonomy: A Scoping Review

**DOI:** 10.7759/cureus.57350

**Published:** 2024-03-31

**Authors:** Yoshiyasu Ito, Rie Oe, Shota Sakai, Yayoi Fujiwara, Hiroshi Kishimoto

**Affiliations:** 1 College of Nursing Art and Science, University of Hyogo, Akashi, JPN; 2 Department of Nursing, Hyogo Prefectural Harima-Himeji General Medical Center, Himeji, JPN

**Keywords:** critical care, scoping review, professional autonomy, nurse, intensive care unit

## Abstract

Intensive care unit (ICU) nurses’ professional autonomy is a critical factor affecting their ability to sustainably provide high-quality care to patients who are critically ill and to their families. However, in the absence of a systematic or scoping review of ICU nurses’ professional autonomy, limited information and evidence are available on this topic. The aim of this scoping review was to clarify the extent and type of evidence on ICU nurses’ professional autonomy. This scoping review was conducted in accordance with the Joanna Briggs Institute methodology for scoping reviews. The following research questions were addressed: (1) Which areas of interest and trends regarding ICU nurses’ professional autonomy have been explored in studies published in scientific journals? And (2) What is known about ICU nurses’ professional autonomy? The data sources included MEDLINE, CINAHL Ultimate, PsycINFO, Cochrane Library, and Ichushi-Web of the Japan Medical Abstracts Society databases. Identified studies were mapped based on their aim, design, methodology, and key findings and categorized according to their focus areas. Of the 734 identified studies, 16 were analyzed. The identified categories were as follows: “relationship between professional autonomy and mental issues,” “experiences and processes of exercising professional autonomy,” “relationship between professional autonomy and nurse-physician collaboration,” “relationship between professional autonomy and demographic characteristics,” “concept of professional autonomy,” “barriers to professional autonomy,” and “team approach to improve professional autonomy.” Most studies have focused on the relationship between professional autonomy and mental health issues and nurse-physician collaboration and few included interventions to enable or promote the exercise of professional autonomy, highlighting a research gap. Future research should identify factors that inhibit the professional autonomy of ICU nurses and that can be changed through interventions and should develop educational and organizational change-based interventions to modify the factors.

## Introduction and background

Nurses working in intensive care units (ICUs) play an important role within healthcare teams in providing care to patients. They are responsible for making decisions in caring for patients who are critically ill and are expected to have professional autonomy. Nurses’ professional autonomy is a care aspect that enhances nursing quality and patient safety [1]. Furthermore, high levels of professional autonomy are also associated with improved patient outcomes, and it has been reported that high levels of professional autonomy of nurses at the hospital level are associated with approximately 19% lower odds of 30-day mortality and 17% lower odds of failure to rescue [2].

Additionally, high levels of autonomy increase nurses’ job satisfaction, lower job stress, and enhance cooperation with physicians [3-5]. ICU nurses are exposed to serious occupational stressors associated with caring for patients who are critically ill and their families [6-8]. Therefore, it is important to educate and support nurses to increase their professional autonomy of nurses to ensure high-quality patient care, improve patient outcomes, and maintain nurses’ job satisfaction and mental health throughout the care process.

However, no systematic or scoping review has been conducted on ICU nurses’ professional autonomy and limited information and evidence are available. A preliminary search of MEDLINE, the Cochrane Database of Systematic Reviews, and Joanna Briggs Institute (JBI) Evidence Synthesis did not reveal any existing or ongoing systematic or scoping reviews on the topic. Therefore, the objective of this scoping review was to clarify the extent and type of evidence on ICU nurses’ professional autonomy.

## Review

Material and methods

Study Design

The scoping review was conducted in accordance with the JBI methodology for scoping reviews [9]. It was reported using the Preferred Reporting Items for Systematic Reviews and Meta-Analyses extension for Scoping Reviews (PRISMA-ScR) checklist [10] (see Appendices Table 3). The protocol was registered on the Open Science Framework [11].

Review Question

The objective of this scoping review was to understand the extent and type of evidence related to the professional autonomy of ICU nurses and the two review questions were as follows: (1) which areas of interest and trends regarding ICU nurses’ professional autonomy have been explored by studies published in scientific journals? and (2) what is known about ICU nurses’ professional autonomy?

Eligibility Criteria

Eligibility criteria were determined by adopting the participant, concept, context (PCC) strategy. All previous studies published in English or Japanese before November 10, 2023, were included, and incomplete articles, such as conference abstracts, or those without full-text access were excluded.

Participants: This scoping review included studies in which data were collected from or about nurses, irrespective of their type, including registered and advanced practice nurses.

Concept: Studies focusing on nurses’ professional autonomy were included. Professional autonomy is a developing trait that is achieved based on patient-based competence and self-reliance to develop the best care plan to improve patients’ health through professional decision-making and professional interactions with other team members [12]. In the nursing field, professional autonomy encompasses clinical autonomy, which is strongly related to decision-making associated with patient care, and nurses’ professional autonomy comprises “independence in decision-making” and the “ability to utilize one’s own competence” [13].

Context: The target settings were adult ICUs (i.e., all ICU types including medical and surgical ICUs), excluding pediatric and neonatal ICUs, multiple wards, and non-ICU departments.

Types of sources: All methodological approaches, including quantitative, qualitative, and mixed-methods designs and all types of sources, except commentaries, editorials, frameworks, guidance documents, and conference abstracts were considered.

Search Strategy

An initial limited search was conducted on MEDLINE (PubMed) and CINAHL Ultimate (EBSCO) to identify relevant articles. The text words contained in the titles and abstracts of relevant articles and index terms used to describe the articles were used to develop a strategy to conduct a full search on MEDLINE (PubMed), CINAHL Ultimate (EBSCO), PsycINFO (EBSCO), Cochrane Library, and Ichushi-Web of the Japan Medical Abstract Society databases (see Appendices Table 4). The reference list of all included sources of evidence was screened for additional studies.

Study/Source of Evidence Selection

The citations identified in the database search were collated and uploaded into the Covidence systematic review software (Veritas Health Innovation, Melbourne, Australia) after removing duplicates. After conducting a pilot test, two independent reviewers (YI and RO) screened titles and abstracts to assess their conformance with the inclusion criteria. Potentially relevant sources were retrieved completely and their citation details were imported into the software. Two independent reviewers (YI and RO) assessed the complete texts of selected citations in detail against the inclusion criteria. The reasons for excluding sources were recorded and reported. Any disagreements between the reviewers at any stage of the selection process were resolved through discussion. The results of the search and the study inclusion process were reported using the PRISMA 2020 flow diagram [14].

Data Extraction

Two independent reviewers (YI and RO) used a data extraction tool they had developed to extract data from the retrieved articles. The extracted data included specific details on research aims or purposes, research population and sample size, research designs, and key findings relevant to the review questions. The tool was not modified or revised during data extraction. The extraction forms were included in the registered protocol. Any disagreements between the reviewers were resolved through discussion or consultation with additional reviewers. The original authors were contacted to provide missing or additional data, where required.

Data Analysis and Presentation

Each study was mapped in detail and its aim, design, methodology, and key findings were described. Studies were categorized and mapped according to their focus areas. In addition, when there were more than two studies on a research area, the findings were summarized and evidence was mapped descriptively.

Results

Study Selection

**Figure 1:**
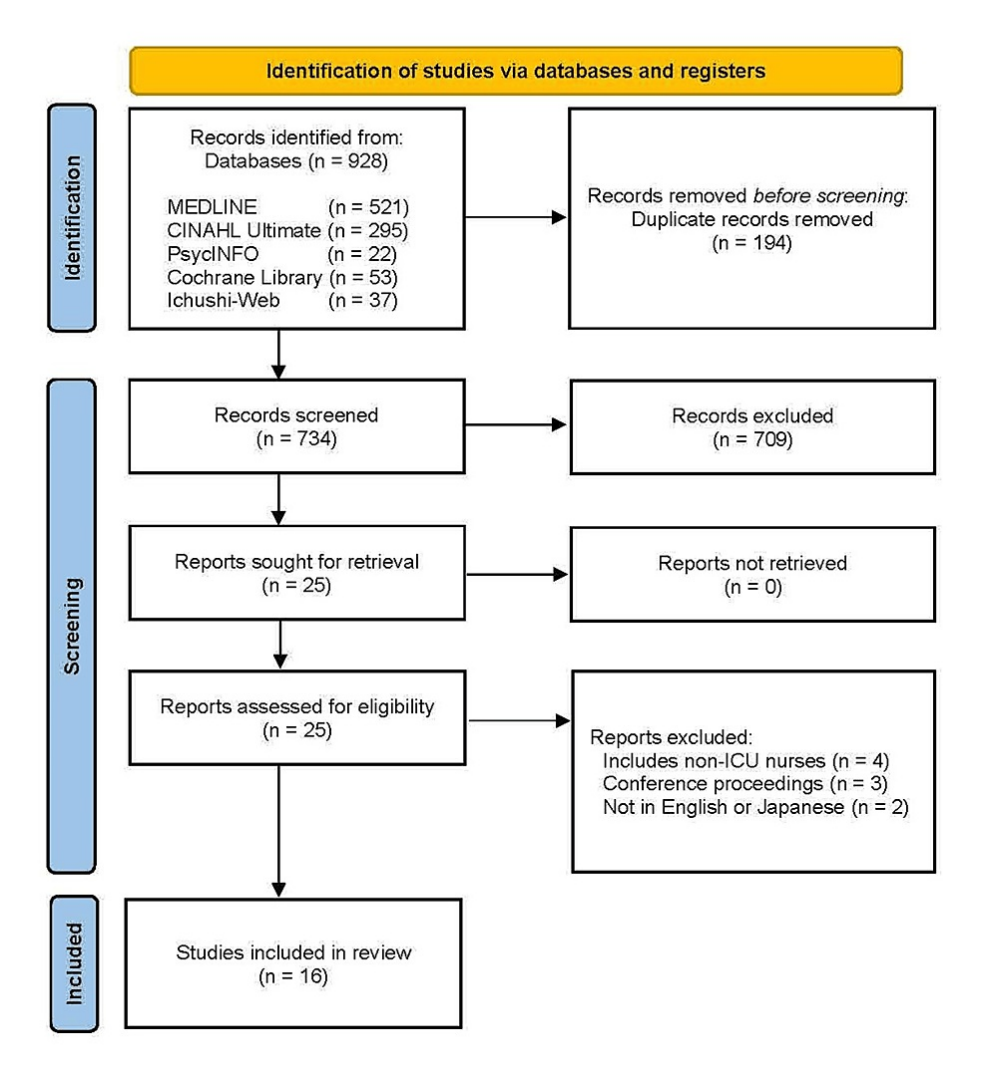
Flowchart of study selection process

After excluding duplicates, 734 studies were identified, of which, 709 were excluded after screening titles and abstracts. Full-text review of the resulting 25 studies was conducted and 16 were selected. The flowchart of the study selection process is presented in Figure 1.

Characteristics of the Studies

Of the 16 identified studies [4,15-29], 15 were published in English [4,15-25,27-29] and one in Japanese [26]. The study designs were as follows: 11 cross-sectional [4,15-18,20,21,23-26], four qualitative [19,22,27,28], and one prospective-comparative [29]. The included studies were published between 2003 and 2023 and conducted in Brazil, Cyprus, Finland, Iran, Italy, Japan, Jordan, the Netherlands, and Poland, with the highest in Iran. Characteristics of the included studies are summarized in Table 1.

**Table 1:** Characteristics of the studies ICU: intensive care unit; VAS: visual analogue scale

Author (year)	Country	Aim	Design	Methodology	Key findings
Costa et al. (2023) [19]	Brazil	To analyze the exercise of professional autonomy of intensive care nurses in the COVID-19 pandemic scenario.	Qualitative	Semi-structured interviews were conducted with 19 ICU nurses, and content analysis in a thematic modality, guided by Eliot Freidson’s Sociology of Professions, was employed.	Amidst the pandemic, it was difficult for nurses to act considering all the prerogatives assigned to them by their social mandate for various reasons, such as limited knowledge about the disease, fragile teamwork and communication, and scarcity of material and human resources.
Taleghani et al. (2023) [27]	Iran	To analyze the concept of autonomy of nurses in ICUs.	Qualitative	The hybrid model approach, as proposed by Schwartz-Barcott and Kim and consisting of theoretical, fieldwork, and analytical phases, was employed. During the fieldwork, semi-structured interviews were conducted with eight ICU nurses.	The antecedents of the concept of nurses’ autonomy in ICUs were: empowerment of the workforce, organizational platform, and social and individual views of the profession. Its attributes were professionalism and high personal capabilities. Finally, increased personal competencies, promotion of quality of care, improved attitudes towards the profession, and professional outcomes were the consequences.
Asl et al. (2022) [15]	Iran	To investigate professional autonomy and its relationship with job stress among nurses working in ICUs.	Cross-sectional	A questionnaire measuring nurses’ personal and social information, professional autonomy, and work-related stress was used. Nurses’ professional autonomy was assessed using the Dempster Practice Behaviors Scale, while work-related stress was measured using the Health and Safety Executive Indicator Tool. Data were collected from 398 ICU nurses, with a response rate of 79.6%.	The mean total professional autonomy and job stress scores of nurses were 102.91±11.88 (range: 30-150), and 115.53±12.42 (range: 35-175), respectively, indicating moderate autonomy and job stress. Professional autonomy had a significant positive correlation with job stress (r = 0.51, p < .00) and autonomy was a significant positive predictor of job stress.
Lis et al. (2022) [20]	Poland	To describe the practices used by nurses to ensure good-quality sleep for adult patients in ICU and assess nurses’ perceptions of patients’ sleep quality and own professional autonomy in sleep management.	Cross-sectional	A questionnaire measured sleep quality assessment, strategies for improving sleep, professional autonomy of nurses in sleep management, and nurse-assessed sleep quality of patients. Data were collected from 119 ICU nurses, with a response rate of 51.3%.	Nurses’ professional autonomy regarding sleep management was average (4.34±2.43, range: 0-10) and correlated with the patient's sleep quality (ρ= 0.25, P < .01). Nurses who rated their autonomy in patients’ sleep management more highly (rho = 0.29, P < .01) and more often influenced patients’ sleep decisions (ρ= 0.24, P < .01) used more methods to improve patients’ sleep.
Parizad et al. (2021) [4]	Iran	To determine nurses’ job stress and its relationship with professional autonomy and nurse-physician collaboration in intensive care unit.	Cross-sectional	A questionnaire was used to measure demographic characteristics, professional autonomy, nurses’ attitudes towards interprofessional collaboration, and work-related stress. Professional autonomy was assessed using the Dempster Practice Behaviors Scale, nurses’ attitudes towards interprofessional collaboration were measured with the Jefferson Scale of Attitudes toward Physician-Nurse Collaboration, and work-related stress was evaluated using the Health and Safety Executive Indicator Tool. Data were collected from 398 ICU nurses, with a response rate of 79.6%.	The mean job stress (115.53±12.42, range: 35-175) and professional autonomy (102.19±11.88, range: 30-150) of ICU nurses were moderate and nurse-physician collaboration (47.53 ± 5.10, range: 15-60) was good. Professional autonomy positively correlated with job stress (r = .506) and nurse-physician collaboration (r = .242). Professional autonomy (p < .00) and work experience in ICU (p = .024) predicted nurses’ job stress.
Aghamohammadi et al. (2019) [24]	Iran	To determine the nurse-physician collaboration and professional autonomy of intensive care nurses.	Cross-sectional	A questionnaire was used to measure professional autonomy and physician-nurse collaboration. Professional autonomy was assessed using the Dempster Practice Behaviors Scale and physician-nurse collaboration was assessed using the Jefferson Scale of Attitudes toward Physician-Nurse Collaboration. Data were collected from 126 ICU nurses, with a response rate of 84.0%.	The mean nurse-physician collaboration score was 47.83 ± 3.9 (range: 15-60), indicating a good collaboration in the ICUs. The results showed that 73% of nurses reported a moderate autonomy and 27% of them considered their autonomy to be high. There was no significant relationship between the nurse-physician collaboration and professional autonomy of the nurses (p >.05).
Yeganeh et al. (2019) [16]	Iran	To determine the relationship between professional autonomy and moral distress of ICU nurses.	Cross-sectional	A questionnaire was used to assess demographic and job characteristics, professional autonomy, and moral distress. Professional autonomy and moral distress were measured using questions employed in previous studies. Data were collected from 180 ICU nurses, with a response rate of 90.0%.	Most respondents were women (93.89%), full-time nurses (61.67%), with a mean age of 35±5.97. Mean professional autonomy and moral distress were 77.04±4.00 (range: 18-108) and 140.85±5.45 (range: 0-288), respectively. Moral distress of most nurses (55.6%) was moderate. There was a positive and significant correlation between professional autonomy and moral distress scores (r=0.33, p<.00)
AllahBakhshian (2017) [28]	Iran	To explore perceived barriers to practice professional autonomy from the perspectives of ICU nurses in Iran.	Qualitative	Semi-structured in-depth interviews were conducted with 28 ICU nurses and the data were analyzed using content analysis.	The following two main themes and five subthemes were identified: (a) profession-related barriers with two subthemes - “lack of capacity to exercise autonomy” and “lack of strong professional bodies” and (b) organizational barriers with three subthemes - "role ambiguity,” “a directive rather than supportive workplace,” and “lack of motivation.”
Georgiou et al. (2017) [25]	Cyprus	To explore nurse-physician collaboration and potential associations with nurses’ autonomy and pertinent nurses’ characteristics in adult ICUs in Cyprus.	Cross-sectional	A questionnaire was used to measure nurse-physician collaboration and autonomy was used. The Collaboration and Satisfaction About Care Decisions Scale was used to measure nurse-physician collaboration. Data were collected from 163 ICU nurses, with a response rate of 88.6%.	The mean score for nurse-physician collaboration was 36.36±13.30 (range: 7-70) and for professional autonomy was 76.15±16.84 (range: 18-108). Higher degree of nurse-physician collaboration associated with higher professional autonomy (r = 0.51, p < ·0001).
Luiking et al. (2017) [29]	Netherlands	To examine the changes in clinical autonomy and in personal norms and values for a planned change and emergent change implementation of an innovation (e.g., intensive insulin therapy).	Prospective comparative	Nurses working in an ICU were randomly assigned to two teams in an experimental condition to compare changes in professional autonomy in a “planned change approach” and an “emergent change approach” in team learning during implementation of intensive insulin therapy. The Nursing Activity Scale was used to assess professional autonomy for each team member pre- and post-intensive insulin therapy implementation.	Pre-implementation measurements did not differ. Post-implementation, clinical autonomy was increased in the emergent change team and decreased in the planned change team. The Personal Values and Norms instrument showed in the emergent change team a decreased hierarchic score and increased developmental and rational scores. In the planned change team, the hierarchical and group scores were increased. Learning did not differ between the teams.
Maharmeh (2017) [21]	Jordan	To describe Jordanian critical care nurses’ experiences of autonomy in their clinical practice.	Cross-sectional	A questionnaire was used to collect demographics and opinions regarding autonomy. Opinions regarding autonomy were measured using an 18-item questionnaire consisting of three domains: knowledge, behavior, and value. Data were collected from 110 ICU nurses, with a response rate of 73.4%.	Cumulative autonomy scores were moderate, exhibiting a mean of 66.58±10.44 (range: 18-108). The highest average autonomy was observed with regard to the scoring of the action base. Female nurses consistently tended to rate their perceived autonomy higher than male nurses, and nurses with more than 10 years of experience had more autonomy (p = .045). The result showed no relationship between yearly income and the autonomy.
Paganini & Bousso (2015) [22]	Brazil	To understand the process by which nurses exercise autonomy in making end-of-life decisions in intensive care units.	Qualitative	Symbolic interactionism and Corbin and Strauss’s grounded theory methodology were used, and semi-structured interviews were conducted with 14 ICU nurses.	Nurses experience the need to exercise autonomy in ICUs on a daily basis. Their experience of increased opportunities to exercise autonomy is conditioned by the pressure of the ICU environment, in which nurses can grow, feel empowered, and exercise their autonomy or continuously depend on others’ decisions.
Karanikola et al. (2014) [17]	Italy	To explore the level of moral distress and potential associations between moral distress indices and (1) nurse-physician collaboration, (2) autonomy, (3) professional satisfaction, (4) intention to resign, and (5) workload among Italian ICU nurses.	Cross-sectional	A questionnaire measuring demographic, educational, and vocational data, moral distress experience, nurses’ autonomy, and nurse-physician collaboration was used. Moral distress experience was measured using Carley's Moral Distress Scale, nurses’ autonomy was measured using the VAS scale, and nurse-physician collaboration was measured using Collaboration and Satisfaction About Care Decisions Scale. Data were collected from 575 ICU nurses, with a response rate of 90.2%. Of them, 566 were used in the analysis, excluding nine incomplete responses.	The mean overall autonomy score (83.5±14.7, range: 18-108) and subscale scores of knowledge (mean = 27.2±5.3, range: 6-36), action (mean = 28.2±5.1, range: 6-36) and value (mean= 27.9±6.6, range: 6-36) base of autonomy were above moderate. The frequency of experiencing moral distress negatively associated with knowledge base of autonomy (r = -0.134, P = 0.004) scores. The overall autonomy score was not associated with moral distress.
Papathanassoglou et al. (2012) [18]	European countries	To explore levels of autonomy among European critical care nurses and potential associations between autonomy and nurse-physician collaboration, moral distress, and nurses’ characteristics.	Cross-sectional	A questionnaire measuring demographics, nurses’ autonomy, nurse-physician collaboration, and moral distress was used. Nurses’ autonomy was measured using an 18-item questionnaire consisting of three domains: knowledge, behavior, and value. Nurse-physician collaboration was measured using the Collaboration and Satisfaction About Care Decisions Scale, and moral distress was measured using the modified Corley Moral Distress Scale. Data were collected from 255 ICU nurses from 17 countries.	The mean autonomy score (84.26±11.7 range: 18-108) was moderate. Autonomy scores were associated with nurse-physician collaboration scores (P< .001) and with a higher frequency of moral distress (P = .04). Autonomy and work satisfaction were associated (P = .001). Frequency of moral distress was associated inversely with collaboration (r = -0.339; P< .001) and autonomy (r = -0.210; P= .01) and positively with intention to quit (r = 0.257; P = .004).
Ominato (2011) [26]	Japan	To investigate the relationship between professional autonomy and years of clinical experience among ICU nurses.	Cross-sectional	A questionnaire measuring demographics and professional autonomy was used. Professional autonomy was assessed using a 47-item questionnaire consisting of five domains: cognition, performance, concrete judgment, abstract judgment, and independent judgment. Data were collected from 175 ICU nurses, with a response rate of 71.1%.	Scores of each of the five domains of professional autonomy and age, years of nursing experience, and years of ICU experience (p<0.01) were correlated.
Varjus et al. (2003) [23]	Finland	To describe Finnish ICU nurses’ experiences of autonomy in their work.	Cross-sectional	A questionnaire measuring demographic data and autonomy issues was used. Autonomy issues were measured using an 18-item questionnaire consisting of three domains, knowledge, action, and value, which was originally developed by the authors. Data were collected from 172 ICU nurses, with a response rate of 65.0%.	Autonomy was composed of three bases: knowledge base (independence, right and responsibility in decision-making), action base (independence, right and responsibility in actions), and value base (independence, right and responsibility in values). Based on the comparisons between the three summed variables, the ICU nurses enjoyed the strongest autonomy in value base (P< 0.003). The majority of the respondents felt they had more autonomy in decision-making and actions concerning patient care than in decision-making and actions concerning the ICU as a whole.

Focus Areas

The identified studies were grouped based on the following categories: “relationship between professional autonomy and mental issues” [4,15-18], “experiences and processes of exercising professional autonomy” [19-23], “relationship between professional autonomy and nurse-physician collaboration” [4,18,24,25], “relationship between professional autonomy and demographic characteristics” [18,21,23,26], “concept of professional autonomy” [27], “barriers to professional autonomy” [28], and “team approach to improve professional autonomy” [29].

Types of Evidence

The areas on which two or more studies focused were “relationship between professional autonomy and mental issues,” “experiences and processes of exercising professional autonomy,” “relationship between professional autonomy and nurse-physician collaboration,” and “relationship between professional autonomy and demographic characteristics.” Several studies showed that the majority of ICU nurses exercised their professional autonomy in decision-making and took appropriate action in their clinical settings [21,23]. Moreover, moral distress, job stress, nurse-physician collaboration, sex, ICU working experience, educational level, continuing education, job independence, and status appraisal were associated with professional autonomy in ICU nurses [4,15-18,23-26]. A descriptive summary of the evidence on each focus area is presented in Table 2.

**Table 2:** Summary of evidence on ICU nurses’ professional autonomy ICU: intensive care unit ^a^ Some studies include more than one focus area.

Focus areas	Number of studies^a^	Evidence
Relationship between professional autonomy and mental issues	5	Three studies showed that professional autonomy is negatively correlated with moral distress at work [16-18], while one showed that professional autonomy and composite moral distress increased when controlling for the effects of educational level, previous ICU experience, and patient-to-nurse ratio [18]. Two studies showed that professional autonomy positively correlated with job stress [4,15].
Experiences and processes of exercising professional autonomy	5	Two studies showed that the majority of ICU nurses exercised professional autonomy in their decision-making and acted in their clinical settings accordingly and that they actualized the action base of autonomy more than the knowledge base of it [21,23]. However, one study showed that ICU nurses had difficulty exercising their professional autonomy during the COVID-19 pandemic due to various factors, including limited knowledge of the disease, weak teamwork, and lack of material and human resources [19]. Two studies showed the exercise of professional autonomy in the specific contexts of “sleep management,” and “end-of-life care” [20,22]. One study showed that the nurses who highly rated professional autonomy in patients’ sleep management and more often influenced decisions regarding patients’ sleep used more strategies to improve patients’ sleep [20]. One study showed that nurses experience the need to exercise autonomy in end-of-life decision-making in ICUs on a daily basis and reported the experiences of “ICU nurses work in a high-pressure environment,” “empower themselves to make decisions,” and “review the spaces where they can exercise autonomy” in the process of increasing opportunities to exercise autonomy [22].
Relationship between professional autonomy and nurse-physician collaboration	4	Three studies showed that professional autonomy is positively correlated with nurse-physician collaboration [4,18,25], whereas one showed that it was not significantly correlated [24].
Relationship between professional autonomy and demographic characteristics of ICU nurses	4	Two studies showed that professional autonomy is higher with more ICU working experience [18,23,26], and Papathanassoglou et al. [18] showed that knowledge base of autonomy was positively correlated with ICU working experience. One study showed no sex differences in the knowledge base of professional autonomy; however, female nurses were more autonomous than their male counterparts in the action and value base, and income did not correlate with professional autonomy [21]. One study showed significant associations between knowledge base of autonomy and educational level, continuing education, job independence, and status appraisal [18].

Discussion

To the best of our knowledge, this is the first study to clarify the extent and type of evidence on ICU nurses’ professional autonomy.

The first important finding of this study was that the most focused areas regarding ICU nurses’ professional autonomy in existing literature were the relationship between professional autonomy and mental health issues. Previous studies have shown that professional autonomy is associated with moral distress and stress even among nurses working in non-ICU settings [5,30,31], suggesting that autonomy is one of the moderators in the relationship between nurses’ work stressors and mental health [5]. In particular, nurses working in ICUs have a higher psychological burden and higher prevalence of mental health issues than nurses working in other areas [8,32,33]. Consequently, many studies have been conducted to develop strategies to prevent mental health issues in ICU nurses [34,35], making it a significant area of research. While there are a variety of work-related stressors in the environment surrounding ICU nurses and finding strategies to eliminate or mitigate them is important, professional autonomy is an individual factor for nurses that mitigates the mental health impact of these stressors, suggesting that it is becoming an important individual factor to focus on in ICU nurses’ mental health measures.

In addition, the findings showed that several studies have highlighted an association between ICU nurses’ professional autonomy and nurse-physician collaboration. Effective collaborations among all stakeholders can benefit organizations, professionals, and patients [36], and nurse-physician collaboration in ICUs influences patient outcomes [37,38]. Therefore, effective nurse-physician collaboration is critical in the ICU and strategies are needed to achieve this. A factor affecting nurse-physician collaboration is the understanding of professional roles; otherwise, nurses will inevitably experience a lack of autonomy, limiting the effectiveness of nurse-physician collaboration [39]. Therefore, for effective nurse-physician collaboration, physicians and nurses need to understand each other’s professional roles; hence, most studies have been conducted on this topic. The evidence suggests that the ability of ICU nurses to exercise professional autonomy can improve patient outcomes by enhancing effective nurse-physician collaboration.

The second important finding is that while studies have emphasized the importance of exercising ICU nurses’ professional autonomy, few have examined interventions to enable or facilitate the exercising of professional autonomy, which is a gap in the literature. This study reveals that previous research has identified several factors associated with ICU nurses’ professional autonomy, many of which, such as demographic characteristics, are not modifiable. However, a study that examined the barriers to professional autonomy perceived by ICU nurses has highlighted the lack of education and training on professional autonomy, a modifiable factor, as an impediment to ICU nurses’ autonomy and its important role in developing nursing identity [28]. Additionally, individual nurses’ profession-related barriers are not the only factors at play, organizational barriers such as existing top-down management style and lack of intra- and inter-professional support [28] are also important. The only intervention study to enhance ICU nurses’ professional autonomy has reported that ICU nurses’ professional autonomy increased by involving team members in planning emergent changes, rather than by implementing already-planned innovative ideas [29]. To fill the research gap in the study of ICU nurses’ professional autonomy, future research needs to develop educational and organizational change-based interventions to modify these individual and organizational factors that inhibit the professional autonomy of ICU nurses.

This study has some limitations. First, as it did not conduct a risk of bias assessment of the identified studies, the reliability of the evidence is uncertain and recommendations regarding clinical judgment and decision-making cannot be made. Second, as studies published in English or Japanese were included and those in other languages were not, the extent and type of evidence may be biased. Third, the limited number of identified studies did not allow the analysis of differences in research trends by country or region, and the extent and type of evidence for cultural context are unknown.

## Conclusions

In this scoping review, we identified the extent and type of evidence on ICU nurses’ professional autonomy. The relationship between professional autonomy and mental health issues and nurse-physician collaboration is the most focused area of research, and studies examining interventions that enable or promote professional autonomy are lacking, highlighting a research gap. Future research should explore modifiable factors that inhibit the professional autonomy of ICU nurses and develop educational and organizational change-based interventions accordingly.

## Appendices

**Table 3:** Preferred Reporting Items for Systematic reviews and Meta-Analyses extension for Scoping Reviews (PRISMA-ScR) checklist JBI: Joanna Briggs Institute; PRISMA-ScR: Preferred Reporting Items for Systematic reviews and Meta-Analyses extension for Scoping Reviews * Where sources of evidence (see second footnote) are compiled from, such as bibliographic databases, social media platforms, and Web sites. † A more inclusive/heterogeneous term used to account for the different types of evidence or data sources (e.g., quantitative and/or qualitative research, expert opinion, and policy documents) that may be eligible in a scoping review as opposed to only studies. This is not to be confused with information sources (see first footnote). ‡ The frameworks by Arksey and O’Malley (6) and Levac and colleagues (7) and the JBI guidance (4, 5) refer to the process of data extraction in a scoping review as data charting. § The process of systematically examining research evidence to assess its validity, results, and relevance before using it to inform a decision. This term is used for items 12 and 16 instead of "risk of bias" (which is more applicable to systematic reviews of interventions) to include and acknowledge the various sources of evidence that may be used in a scoping review (e.g., quantitative and/or qualitative research, expert opinion, and policy document). From Reference [10]

SECTION	ITEM	PRISMA-ScR CHECKLIST ITEM	REPORTED ON PAGE #
TITLE			
Title	1	Identify the report as a scoping review.	p.1
ABSTRACT			
Structured summary	2	Provide a structured summary that includes (as applicable): background, objectives, eligibility criteria, sources of evidence, charting methods, results, and conclusions that relate to the review questions and objectives.	p.1
INTRODUCTION			
Rationale	3	Describe the rationale for the review in the context of what is already known. Explain why the review questions/objectives lend themselves to a scoping review approach.	p.1
Objectives	4	Provide an explicit statement of the questions and objectives being addressed with reference to their key elements (e.g., population or participants, concepts, and context) or other relevant key elements used to conceptualize the review questions and/or objectives.	p.1
METHODS			
Protocol and registration	5	Indicate whether a review protocol exists; state if and where it can be accessed (e.g., a Web address); and if available, provide registration information, including the registration number.	p.1,2
Eligibility criteria	6	Specify characteristics of the sources of evidence used as eligibility criteria (e.g., years considered, language, and publication status), and provide a rationale.	p.2
Information sources*	7	Describe all information sources in the search (e.g., databases with dates of coverage and contact with authors to identify additional sources), as well as the date the most recent search was executed.	p.2
Search	8	Present the full electronic search strategy for at least 1 database, including any limits used, such that it could be repeated.	Appendices
Selection of sources of evidence†	9	State the process for selecting sources of evidence (i.e., screening and eligibility) included in the scoping review.	p.2
Data charting process‡	10	Describe the methods of charting data from the included sources of evidence (e.g., calibrated forms or forms that have been tested by the team before their use, and whether data charting was done independently or in duplicate) and any processes for obtaining and confirming data from investigators.	p.2,3
Data items	11	List and define all variables for which data were sought and any assumptions and simplifications made.	p.2
Critical appraisal of individual sources of evidence§	12	If done, provide a rationale for conducting a critical appraisal of included sources of evidence; describe the methods used and how this information was used in any data synthesis (if appropriate).	N/A
Synthesis of results	13	Describe the methods of handling and summarizing the data that were charted.	p.2,3
RESULTS			
Selection of sources of evidence	14	Give numbers of sources of evidence screened, assessed for eligibility, and included in the review, with reasons for exclusions at each stage, ideally using a flow diagram.	Figure.1
Characteristics of sources of evidence	15	For each source of evidence, present characteristics for which data were charted and provide the citations.	p.3 and Table.1
Critical appraisal within sources of evidence	16	If done, present data on critical appraisal of included sources of evidence (see item 12).	N/A
Results of individual sources of evidence	17	For each included source of evidence, present the relevant data that were charted that relate to the review questions and objectives.	p.7, 8 and Table.1, 2
Synthesis of results	18	Summarize and/or present the charting results as they relate to the review questions and objectives.	p.7, 8 and Table.1, 2
DISCUSSION			
Summary of evidence	19	Summarize the main results (including an overview of concepts, themes, and types of evidence available), link to the review questions and objectives, and consider the relevance to key groups.	p.8,9
Limitations	20	Discuss the limitations of the scoping review process.	p.9
Conclusions	21	Provide a general interpretation of the results with respect to the review questions and objectives, as well as potential implications and/or next steps.	p.9
FUNDING			
Funding	22	Describe sources of funding for the included sources of evidence, as well as sources of funding for the scoping review. Describe the role of the funders of the scoping review.	p.10

**Table 4:** Search strategy

Database	Search strategy	Results
MEDLINE (PubMed)	("Nursing"[MeSH Terms] OR "nurs*"[Title/Abstract]) AND ("Professional Autonomy"[MeSH Terms] OR "clinical autonomy"[Title/Abstract] OR "autonomy"[Title/Abstract]) AND ("Intensive Care Units"[MeSH Terms] OR "Critical Care Nursing"[MeSH Terms] OR "Critical Illness"[MeSH Terms] OR "Critical Care"[MeSH Terms] OR "ICU"[Title/Abstract] OR "intensive care unit*"[Title/Abstract] OR "critically ill*"[Title/Abstract] OR "intensive care"[Title/Abstract])	521
CINAHL Ultimate (EBSCO)	(TI "Nurs*" OR AB "Nurs*") AND ((MH "Professional Autonomy") OR (TI "Clinical Autonomy" OR AB "Clinical Autonomy") OR (TI "Autonomy" OR AB "Autonomy")) AND ((MH "Intensive Care Units") OR (MH "Critical Care Nurses") OR (MH "Critical Illness") OR (MH "Critical Care") OR (TI "ICU" OR AB " ICU") OR (TI "Intensive Care Unit*" OR AB "Intensive Care Unit*") OR (TI "Critically ill*" OR AB "Critically ill*") OR (TI "Intensive Care" OR AB "Intensive Care"))	295
PsycINFO (EBSCO)	((MM "Nursing") OR (DE "Nurses") OR (TI "Nurs*" OR AB "Nurs*")) AND ((TI "Professional Autonomy" OR AB "Professional Autonomy") OR (TI "clinical autonomy" OR AB "clinical autonomy") OR (MM "Autonomy")) AND ((MM "Intensive Care") OR (TI "critical care" OR AB "critical care") OR (MM "Critical Illness") OR (TI "ICU" OR AB " ICU") OR (TI "Intensive Care Unit*" OR AB "Intensive Care Unit*"))	22
Cochrane Library	((MeSH descriptor: [Nursing] explode all trees) OR ((Nurs*):ti,ab,kw (Word variations have been searched))) AND ((MeSH descriptor: [Professional Autonomy] explode all trees) OR ((Clinical Autonomy):ti,ab,kw (Word variations have been searched)) OR ((Autonomy):ti,ab,kw (Word variations have been searched))) AND ((MeSH descriptor: [Intensive Care Units] explode all trees) OR (MeSH descriptor: [Critical Care Nursing] explode all trees) OR (MeSH descriptor: [Critical Illness] explode all trees) OR (MeSH descriptor: [Critical Care] explode all trees) OR ((ICU):ti,ab,kw (Word variations have been searched)) OR ((Intensive Care Unit):ti,ab,kw (Word variations have been searched)) OR ((Critically ill*):ti,ab,kw (Word variations have been searched)) OR ((Intensive Care):ti,ab,kw (Word variations have been searched)))	53
Ichushi-Web of the Japan Medical Abstract Society Databases	(([看護師]/TH) OR ("看護"/TH)) AND (([専門職の自律性]/TH) OR (専門職自律性/TA) OR (専門職的自律性/TA) OR (自律性/TA)) AND (([ICU]/TH) OR ([クリティカルケア]/TH) OR ([ICU看護]/TH) OR ([クリティカルケア看護]/TH))	37
